# Association Between Metabolic Parameters and FTO Alpha-Ketoglutarate-Dependent Dioxygenase (*FTO*), Transcription Factor 7-like 2 (*TCF7L2*), and Solute Carrier Family 16 Member 11 (*SLC16A11*) Alleles in Mexican Children and Adolescents

**DOI:** 10.3390/ijms27135948

**Published:** 2026-07-02

**Authors:** Adriana Díaz-Anzaldúa, José Octavio Hernández-Lagunas, Andrés García-Sibaja, Ilse Mandujano-Ramírez, Alfonso Cabrera Lagunes, Lino Palacios-Cruz, Ana Rodriguez-Ventura

**Affiliations:** 1 Departamento de Genética, Dirección de Investigaciones Biomédicas en Salud Mental, Instituto Nacional de Psiquiatría Ramón de la Fuente Muñiz, Mexico City 14370, Mexico; 2 Dirección de Investigaciones Biomédicas en Salud Mental, Instituto Nacional de Psiquiatría Ramón de la Fuente Muñiz, Mexico City 14370, Mexico; 3 Departamento de Fisiología y Desarrollo Celular, Instituto Nacional de Perinatología Isidro Espinosa de los Reyes, Mexico City 11000, Mexico; 4 Departamento de Embriología y Genética, Facultad de Medicina, Universidad Nacional Autónoma de México, Mexico City 04510, Mexico

**Keywords:** FTO alpha-ketoglutarate-dependent dioxygenase (*FTO*), transcription factor 7-like 2 (*TCF7L2*), solute carrier family 16 member 11 (*SLC16A11*), rs9939609, rs7895307, rs75493593, body mass index, waist circumference, metabolic risk, mental disorders, depression, children, adolescents, homeostatic model assessment for insulin resistance (HOMA-IR index)

## Abstract

Rs9939609 marker in FTO Alpha-Ketoglutarate-Dependent Dioxygenase (*FTO*) gene, rs7895307 in Transcription Factor 7-Like 2 (*TCF7L2*) gene, and rs75493593 in Solute Carrier Family 16 Member 11 (*SLC16A11*) gene have been associated with anthropometric, metabolic, and clinical variables, but have not been concurrently studied in Mexican children and adolescents with adiposity or mental disorders. In this cross-sectional association study, we genotyped these markers by means of TaqMan real-time polymerase chain reaction in two at-risk pediatric cohorts recruited in Mexico City. Group 1 (n = 175) comprised children and adolescents with overweight/obesity. Group 2 (n = 296) consisted of non-medicated adolescents meeting the Diagnostic and Statistical Manual of Mental Disorders, fourth edition criteria for Attention Deficit/Hyperactivity Disorder or a mood disorder. Anthropometric measurements (body mass index —BMI—, waist circumference, body fat percentage), metabolic indices (fasting glucose, lipid profile, Homeostatic Model Assessment for Insulin Resistance), and psychiatric diagnoses were evaluated. In Group 1, the *FTO* A allele (genotypes AA/AT) was significantly associated with severe obesity according to BMI Z scores (*p* = 0.004, O.R. 3.33, 95% CI [1.42–7.77]), and it was a predictor of waist circumference (B = 6.16, 95% CI [1.78–10.55], *p* = 0.006) and muscle percentage (B = 4.21%, 95% CI [0.91–7.51%], *p* = 0.013) using linear regression models adjusted for age and sex. In Group 2, *TCF7L2* AA genotype was associated with increased odds of depression (B = 0.83, *p* = 0.003, OR = 2.29, 95% CI [1.32–3.96]). While *SLC16A11* G allele showed a possible association with insulin resistance or glucose levels, confirmation is needed. These exploratory results highlight the need for larger, well characterized cohort studies to confirm the associations.

## 1. Introduction

Obesity and overweight (adiposity) are currently a major health problem worldwide. In Mexico, according to the National Health and Nutrition Survey, its prevalence increased to 40.1 in adolescents [1]. Obesity in children is an important risk factor for early onset chronic diseases and premature death [2], and it is associated with mental health in adolescents [3,4]. An increase in adiposity is related to a higher cardiovascular risk and a negative impact on child development [3]. In addition, the increase of adipose tissue can cause inflammation, which may contribute to decreased insulin sensitivity and mitochondrial dysfunction [5]. The association between obesity and insulin resistance is related to visceral fat, even in children [6].

In Mexico, type 2 diabetes (T2D) has a prevalence of 18.3%, ranking as the second leading cause of death and the leading cause of disability. The prevalence of T2D has increased in this population, frequently in association with obesity [7]. Furthermore, mental disorders, such as Attention Deficit/Hyperactivity Disorder (ADHD), have been associated with an increased risk of T2D [8].

In addition, the prevalence of dyslipidemia in this country was estimated to be 36.7% [9]. About 20% of a sample of Mexican youth were shown to have metabolic syndrome (abdominal obesity, elevated fasting glucose and blood pressure, and reduced high-density lipoprotein cholesterol—HDL-C), while only 10% were free of any metabolic syndrome component [10]. Metabolic syndrome has also been linked to disorders such as depression [11].

**Figure 1:**
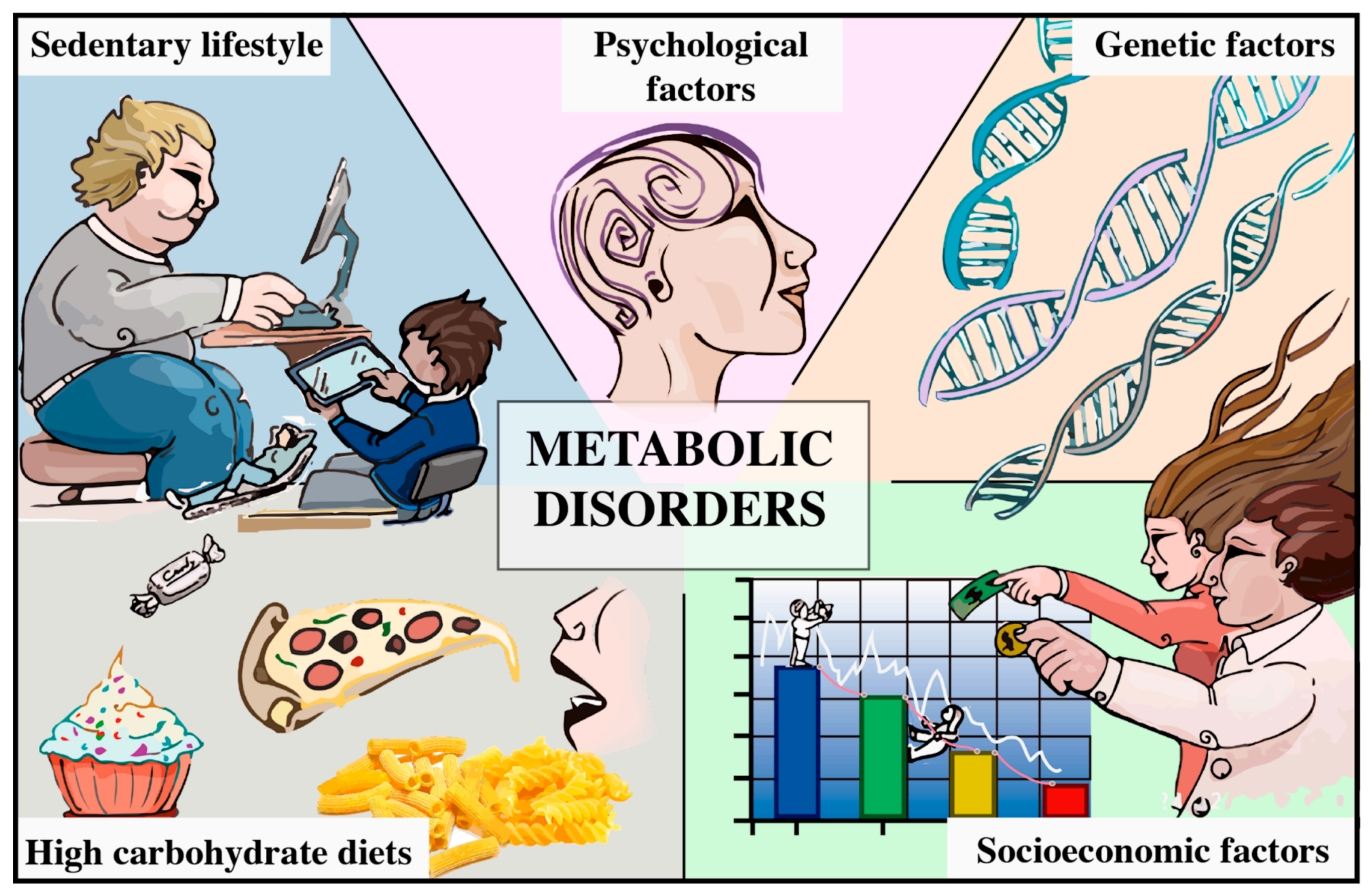
Main risk factors for obesity, diabetes, and dyslipidemia in the pediatric population.

Dyslipidemia, obesity, and T2D develop because of an interaction of various factors such as multiple genetic variants and unhealthy habits, in conjunction with socioeconomic, psychological, and other types of components (Figure 1). Heritability estimates for body mass index (BMI) range from 47% to 90% in twin studies and 24 to 81% in family studies [12]; for T2D, heritability varies between 25% and 80% [13]. An extended phenotype including impaired glucose tolerance at 15 years follow-up showed a concordance of 96% in monozygotic twins [14]. In addition, the risk for T2D could be significantly increased by having a parent or a sibling with T2D [15]. Regarding disorders in lipid metabolism, heritability for dyslipidemia was found to be 42%. For low-density lipoprotein cholesterol (LDL-C), it was estimated to be between 32% and 69%, for HDL-C from 23% to 80%, while for total cholesterol, it ranged from 42% to 67% [16]. In the case of triglycerides, genetic differences may contribute to 50% of the variance in the phenotype [17].

Genome-wide association studies (GWASs) have repeatedly confirmed the association between rs9939609 marker of the FTO Alpha-ketoglutarate-dependent dioxygenase (*FTO*) gene and the risk of overweight, obesity, higher risk of increased Z score, and BMI percentiles in multiple adult and pediatric populations [18]. In Mexico, the link between the *FTO* gene and T2D was shown to be mediated by an association with BMI in 580 children aged 8 to 13 years in the city of Queretaro. Likewise, a significant association was found with obesity and the presence of homozygosity for the A allele of rs9939609 marker. This gene is expressed in the hypothalamus and is associated with pathways related to appetite and energy intake [19]. In addition, in recent years, more than 20 variants of the Transcription factor 7-like 2 (*TCF7L2*) gene have been associated with T2D and high BMI, including rs7895307 and rs12772424 markers [20,21]. In Mexico, the association between *TCF7L2* markers and T2D was replicated in the state of Guerrero and Mexico City [22]. This gene codes for a transcription factor associated with the Wnt signaling pathway, beta cell function, and insulin secretion [23]. Furthermore, it has been observed that one of the potential risk factors for T2D, identified through GWASs, is the rs75493593 polymorphism of the Solute Carrier Family 16 Member 11 (*SLC16A11*) gene, harboring a possibly risk-conferring variant, which is more prevalent among individuals of Mexican or Latin American ancestry than in other groups. In a study, each allele of a haplotype was associated with disease vulnerability. The research showed that people with risk variants presented T2D on average 2.1 years earlier and with lower BMI than people who did not have these variants. *SLC16A11* gene encodes a protein found in a variety of tissues, with higher levels in thyroid, liver, and salivary glands, and it may be involved in lipid metabolism [24].

Given the rapid increase in the prevalence of metabolic conditions and the negative effect they have on health, it is of interest to learn more about possible factors that contribute, at the genetic level, to a greater risk of overweight, obesity, T2D, and cardiovascular disease in children. Genetic variants of *FTO*, *TCF7L2*, and *SLC16A11* have been associated with metabolic phenotypes mainly in populations of European or Asian origin, and to a lesser extent from other ancestries [25,26], yet evidence from Latin American pediatric samples is still scarce. This is important because genetic variants may interact with region-specific environmental exposures (e.g., type of diet and physical activity), and allele frequencies vary among populations. In addition, it is particularly important to increase the study of possible genetic risk factors in populations that are more susceptible to obesity and insulin resistance, such as Latin American, African American, and Native American groups [26], especially in children and adolescents with overweight, obesity, or mental disorders. To date, no studies have been identified in which rs9939609 (*FTO*), rs7895307 (*TCF7L2*), and rs75493593 (*SLC16A11*) have been concurrently analyzed in at-risk Mexican children and adolescents. Moreover, research is needed on the genetic component of an extended metabolic phenotype in Mexican children that considers lipid levels, alterations in liver enzymes, and hormones such as insulin, which contribute to the pathogenesis of T2D, obesity, and cardiovascular disease. The present study aims to investigate the hypothesis that a comprehensive set of anthropometric, metabolic, and psychiatric phenotypes are associated with these three genetic polymorphisms in two at-risk Mexican pediatric samples.

## 2. Results

There were 471 recruited individuals divided in two groups. Group 1 comprised children/adolescents with adiposity (overweight or obesity), including 92 girls and 83 boys, with a mean age of 14.2 and 13.61 years, respectively. Participants in Group 2 sought treatment for mental disorders, including 145 girls and 151 boys, with a median age of 14 and 15 years, respectively. Table 1 and Table 2 describe characteristics of the sample (Groups 1 and 2, respectively).

As Table 3 shows, there was no deviation from Hardy–Weinberg equilibrium in genotype frequencies for *FTO* (rs9939609) and *TCF7L2* (rs7895307) markers, but there was a deviation for *SLC16A11* (rs75493593), mainly in Group 1 (*p* = 0.011).

### 2.1. FTO Alpha-Ketoglutarate-Dependent Dioxygenase (FTO) Gene

Frequencies of rs9939609 marker genotypes were similar in both groups (X^2^ = 0.29, *p* = 0.86). In Group 1, there was an association between the presence of A allele (AA or AT genotypes) and severe obesity according to BMI Z score (Pearson X^2^ = 8.22, *p* = 0.004, O.R. 3.33, 95% CI [1.42–7.77]). Linear regression models including marker rs9939609, sex, and age explained part of the variance of metabolic parameters. As shown in Table 4, the first model explained 6.94% of the variance on BMI Z score in Group 1, with a significant effect (F = 3.66, *p* = 0.014, R^2^ = 0.07). The presence of A allele was a predictor of the BMI Z score (B = 0.45, 95% CI [0.13–0.78], β = −0.22, *p* = 0.007), with no evidence of influence of male sex (*p* = 0.438) or age (*p* = 0.129). These variables also explained 24.02% of the variance of waist circumference (F = 14.22, *p* = <0.001, R^2^ = 0.24). The predictors were male sex (B = 6.63, 95% CI [2.38–10.89], β = 0.22, *p* = 0.002), age (B = 1.59, 95% CI [1.01–2.18], β = 0.38, *p* = <0.001), and the AA/AT genotype (B = 6.16, 95% CI [1.78–10.55], β = 0.20, *p* = 0.006). For fat percentage, the model explained 9.79% of the variance (F = 5.25, *p* = 0.002, R^2^ = 0.10), and the only predictor was being a female (B = 4.04, 95% CI [1.49–6.59], β = 0.25, *p* = 0.002), but not FTO AA/AT genotype (*p* = 0.019) or age (*p* = 0.52). Regarding muscle percentage, 60.52% of its variance was explained (F = 37.82, *p* = <0.001, R^2^ = 0.61). In this case, the predictor variables were male sex (B = 6.00%, 95% CI [2.79–9.21%], β = 0.28, *p* = <0.001), age (B = 2.67%, 95% CI [2.12–3.22%], β = 0.71, *p* < 0.001), and AA/AT genotype (B = 4.21%, 95% CI [0.91–7.51%], β = 0.19, *p* = 0.013). There were no differences in genotype (carriers vs. non-carriers) or allele frequencies in participants with or without insulin resistance according to Homeostatic Model Assessment for Insulin Resistance (HOMA-IR) index (Table 5).

In Group 2, body fat percentage was not associated with rs9939609 marker (Mann–Whitney U = 2803, *p* = 0.24). The regression model in Table 4 explained 29.99% of the variance in fat percentage (F = 23.76, *p* = <0.001, R^2^ = 0.30), and confirmed that a predictor of this model was being a female (B = 9.51, 95% CI [7.21–11.81], β = 0.54, *p* = <0.001), but not the AA/AT genotype (*p* = 0.029) or age (*p* = 0.039).

### 2.2. Transcription Factor 7-like 2 (TCF7L2) Gene

The allelic frequencies of rs7895307 were similar in both groups (χ^2^ = 1.17, *p* = 0.56). As shown in Table 6, a model explained 9.34% of the variance in fasting glucose levels in Group 1 (F = 3.68, *p* = 0.007, R^2^ = 0.09), but there was not enough evidence of specific predictors, GG genotype (*p* = 0.017), age (*p* = 0.02), or sex (*p* = 0.064). Table 5 shows that there was no evidence of association between genotype (GG vs. AA/AG) or allele frequencies (G vs. A) and HOMA-IR (Fisher exact *p* = 0.03 and Pearson *p* = 0.02, respectively).

Regarding Group 2, as shown in Table 6, a model explained 8.25% of the variance in fasting glucose (F = 6.32, *p* < 0.001, R^2^ = 0.08), and it was found that male sex (B = 3.13, 95% CI [1.57–4.70], β = 0.23, *p* < 0.001) predicted glucose levels, but there was no evidence of the influence of GG genotype (*p* = 0.137), GA genotype (*p* = 0.659), or age (*p* = 0.359). When evaluating the presence of depression, a binary logistic regression model explained 12.54% of the variance (X^2^ = 22.86, *p* < 0.001). In this clinical sample, an increased odds of depression was found in women (B = 0.96, *p* < 0.001, OR = 2.61, 95% CI [1.53–4.44]) and in carriers of AA genotype (B = 0.83, *p* = 0.003, OR = 2.29, 95% CI [1.32–3.96]), with no evidence of the effect of glucose (B = 0.02, *p* = 0.378, OR = 1.02, 95% CI [0.98–1.06]) or age (B = 0.13, *p* = 0.156, OR = 1.14, 95% CI [0.95–1.36]).

### 2.3. Solute Carrier Family 16 Member 11 (SLC16A11) Gene

The frequency of the rs75493593 genetic variant did not significantly differ between the two groups (χ^2^ = 2.95, *p* = 0.23). However, within Group 1, this variant was strongly associated with insulin resistance, as measured by the HOMA-IR index. Specifically, individuals with the GG or GT genotypes were nearly five times more likely to exhibit insulin resistance than those with TT genotype (Fisher *p* = 0.0134, OR = 4.82, 95% CI [1.43–16.25]), and the presence of G allele in general was also associated (Pearson χ^2^ = 7.57, OR = 2.37, 95% CI [1.28–4.43], *p* = 0.005) in those with insulin resistance (Table 5). As shown in Table 7, in Group 2, a model showed a moderate positive relationship with fasting glucose values, explaining 9.86% of its variance (F = 8.81, *p* < 0.001, R^2^ = 0.10), where being a male (B = 3.55, 95% CI [2.06–5.03], β = 0.25, *p* < 0.001) or carrier of GG or GT genotype (B = 1.97, 95% CI [0.40–3.54], β = 0.13, *p* = 0.014) were predictors of glucose, with no evidence of an influence of BMI percentile (*p* = 0.024) or age (*p* = 0.179).

## 3. Discussion

This study investigated the association between three genetic polymorphisms—rs9939609 (*FTO*), rs7895307 (*TCF7L2*), and rs75493593 (*SLC16A11*)—and specific metabolic, clinical, and anthropometric traits. We focused on a historically understudied population to address gaps left by research predominantly conducted on European and Asian groups. To reduce phenotypic heterogeneity and enhance our ability to detect biologically meaningful genetic associations, we also analyzed specific phenotypes rather than only broad clinical measurements. These included extreme obesity, waist circumference, HOMA-IR index, muscle percentage, and mental disorders. Our study focused on two pediatric cohorts from Mexico City—an area with above-average childhood obesity and sedentarism [27]. Group 1 included at-risk youth with adiposity; Group 2 included youth with mental disorders. This specific selection of participants seeks to maximize the probability of detecting relevant genetic associations with the metabolic phenotypes under study. We propose the hypothesis that a stronger association between these markers could be present when studying at-risk children, as opposed to adults from the general population, which is the most studied group. In this case, an increased statistical power could be reached in the detection of small effect variants, even with moderate sample sizes.

The A allele of the rs9939609 marker in the *FTO* gene has been associated with increased BMI and obesity in adult and pediatric populations [26,28]. In Group 1, this allele was associated with BMI Z score, controlling for age and sex, and with extreme- or high-risk phenotypes: severe obesity according to BMI Z score, and waist circumference. The latter mainly represents visceral fat, associated with chronic inflammation, insulin resistance, and cardiovascular disease. If *FTO* is associated with central obesity, its role in cardiometabolic risk may be even higher. BMI has the advantage of being measured in large samples worldwide. However, relying solely on this variable to assess metabolic health is limited. It cannot differentiate between fat mass and lean muscle mass, nor does it account for natural variations in fat distribution and skeletal structure, particularly across understudied populations. Our results suggest that, upon validation, some extreme phenotypes may enhance our ability to stratify genetic metabolic risk. When evaluating this polymorphism along with age and sex, the presence of A allele was also a predictor of muscle percentage, which has not been described in the Mexican population. It has been proposed that FTO has a function beyond adipose tissue or the central nervous system, associating this gene with skeletal muscle differentiation. A high expression of FTO protein has been observed not only in adipose tissue, but also in skeletal muscle [29]. We found that carriers of A allele had four times more muscle percentage than non-carriers. Research indicates that patients with greater muscle mass often exhibit two distinct traits. First, they have higher C-peptide levels, which may serve as a compensatory mechanism to maintain stable glucose levels. Second, they present with lower HDL-cholesterol levels, which is a major risk factor for cardiovascular disease [30]. No genetic association was found in Group 2. While participants were not medicated, it will be important to evaluate in this group of adolescents other clinical variables, such as specific psychiatric diagnoses and comorbidity, clinical subgroups, and lifestyle, among other factors.

For rs7895307 marker in the *TCF7L2* gene, in Group 1 there was no association with glucose or other metabolic parameters. Given that the results were not far from being significant, it may be important not to discard the possibility of a non-detected association and to study larger samples and possibly haplotypes at *TCF7L2*. In Group 2, there was also no evidence of association with glucose levels. However, the genotype, in this case AA, and being a woman were predictors of depression. *TCF7L2* has been identified as a shared loci for diabetes and depressive symptoms, and markers in this gene may be associated with a risk for either depression (rs12255179) or diabetes (rs61872794) [31,32]. While our result suggest that it is at least twice as likely to have depression for women or AA genotype carriers, further studies with larger samples could investigate the hypothesis of an association between depression and *TCF7L2* in the Mexican population and worldwide. TCF7L2 is expressed in mouse and human astrocytes, especially during astrocyte maturation, and it has been associated with social interaction in mice [33]. If the association is confirmed, one possible explanation to evaluate could be that astrocytes regulate different functions in the nervous system, and they have been linked to depression [34].

Finally, the rs75493593-G allele at *SLC16A11* was twice as likely to occur in the group with insulin resistance in Group 1. It was also a predictor of fasting glucose levels in Group 2, along with sex. These exploratory results should be further investigated. The frequency of T allele in Mexico and other Latin American populations is one of the highest in the world [35,36]. T allele, rather than G, has been associated with glucose or insulin levels in Latin American samples, so our result of a possible association with G allele remains controversial. About half of the sample in Group 1 had missing information for HOMA-IR. In addition, the lack of Hardy–Weinberg equilibrium could be associated with genotyping errors, the effect of a small sample size, population stratification, or selection bias due to a non-probabilistic recruitment, among other factors. We re-genotyped 12% of the samples and two of us independently confirmed the genotypes. Every assay included at least two negative and three positive controls. We found an underrepresentation of heterozygotes in both groups (n = 64 when the expected was 73 in Group 1 and n = 148 when the expected was 171 in Group 2). This underrepresentation is also found in samples of volunteers of Mexican Origin in Los Angeles California [36], and a lack of Hardy–Weinberg equilibrium is also shown in a sample of American Indians from the Phoenix Study on Nephropathy and Diabetes [37]. Future studies could be performed to investigate our results and confirm genotyping by sequencing.

Among the limitations we faced are the small sample size, non-probabilistic sampling, missing data in several anthropometric or metabolic variables, the fact that the two groups were recruited from different institutions, over different periods, had distinct clinical background, and not all metabolic variables were measured in both samples. Group 1 was not assessed for mental disorder, and Group 2 for parameters such as insulin or glycated hemoglobin. For that reason, our analyses of Groups 1 and 2 were independent. The lack of analysis of variables such as diet and physical activity was also important. Further research in diverse populations is critical to confirm and extend these findings.

Our study underscores the importance of increasing the number of genetic investigations in non-European or Asian populations, taking into consideration allelic frequencies around the world, and the need for studying gene–environment interactions in complex phenotypes such as obesity, and other metabolic conditions. We propose that utilizing extreme, high-risk, or comprehensive phenotypes, rather than broad metabolic variables, may improve the detection of true, biologically relevant genetic associations. Ultimately, integration of genetic data with well-characterized, large-scale samples—encompassing mental health, socioeconomic, and lifestyle data—could help generate or confirm stronger hypotheses.

## 4. Materials and Methods

A total of 471 adolescents were consecutively recruited through non-probabilistic sampling. This cross-sectional association study included two groups recruited from different institutions, over different time periods. In Group 1, children or adolescents with adiposity according to BMI Z score were recruited at a tertiary-level medical institution in Mexico City, between 2019 and 2020, to participate in the “Sacbe” comprehensive program to decrease their adiposity [38]. Exclusion criteria were being on treatment with metformin, steroids, or other substance affecting glucose, and having a disabling disorder that would prevent them from exercising. In Group 2, adolescents were recruited at another tertiary-level medical institution in the same city, from 2008 to 2016; they were first-time visiting patients, non-medicated, who met the DSM-IV criteria for ADHD and/or mood disorder, assessed by a certified psychiatrist with at least fifteen years of clinical experience. Exclusion criteria included having a neurological or personality disorder, intellectual disability, schizophrenia, bipolar disorder, or any severe psychiatric condition at recruitment. In both groups, anthropometric variables, 12 h fasting glucose and lipid profile (total cholesterol, triglycerides, HDL-cholesterol, LDL-cholesterol), uric acid, as well as fat percentage were evaluated. In Group 1, participants were also evaluated for glycated hemoglobin (HbA1c), alanine aminotransferase (ALT), aspartate aminotransferase (AST), gamma-glutamyl transferase (GGT), insulin, and C-peptide, but not for mental disorders or blood pressure. In Group 2, participants were evaluated for systolic and diastolic blood pressure, but not for insulin, C-peptide, HbA1c, or liver enzymes.

A body composition scanner was used to measure body weight, body fat, and body muscle, and a stadiometer for height in participants of both groups. For waist circumference, participants were asked to stand upright in a comfortable position, with feet together and arms relaxed at their sides, in the presence of one of their parents. Just after a normal exhalation, a metal tape was used to measure waist (4 cm above umbilicus) two times, against bare skin, keeping the tape parallel to the floor. The mean value of both measurements was registered. BMI Z score ≥3 was considered as severe obesity (+3 standard deviations).

In Group 1, HOMA-IR index was calculated using fasting glucose (mg/dL) × fasting insulin (µU/mL)/405. Patients with HOMA-IR values ≤ 2.8 were considered within the normal range, while those with HOMA-IR ≥ 2.9 were classified as insulin resistance.

For children 7 to 9 years old, triglycerides were considered healthy (<75 mg/dL), borderline high (75–99 mg/dL), or high (≥100 mg/dL). For 10- to 19-year-olds, they were considered healthy (<90 mg/dL), borderline high (90–129 mg/dL), or high (≥130 mg/dL). Total cholesterol was categorized as healthy (<170 mg/dL), borderline high (170–199 mg/dL), and high (≥200 mg/dL). LDL-cholesterol, healthy (<110 mg/dL), borderline high (110–129 mg/dL), and high (≥130 mg/dL). HDL-cholesterol, healthy (>45 mg/dL), borderline low (40–45 mg/dL), and low (<40 mg/dL). For fasting glucose, serum glucose was evaluated in patients with normal (<100 mg/dL) and borderline (100–125 mg/dL) values, based on the American Diabetes Association recommendations for diagnosing prediabetes.

A 5 mL blood sample was obtained from each person, from which DNA was extracted using the FlexiGene DNA kit (250) (Qiagen N.V., Hilden, North Rhine-Westphalia, Germany). To determine DNA concentration and purity, a Nanodrop 2000 spectrophotometer (Thermo Fisher Scientific, Waltham, MA, USA) was used, evaluating absorbance at 230, 360, and 280 nm, with 260/280 nm ratio close to 1.8. Real-time PCR was performed using Thermo Fisher Scientific TaqMan Assays in a 7500 Real Time PCR System (Applied Biosystems, Waltham, MA, USA). A test was performed for each marker. PCR conditions were confirmed and three samples, each of a different genotype, were chosen as future positive controls. For each 96-well plate assay, at least two no-template control wells were included, and the assay was considered only if there was no amplification in these wells. The positive controls were also included in each plate. For allele calling, homozygous samples had to fall close to their relative x- or y-axis, and heterozygous midway between the other two clusters. The quality value of the software was set to 0.95, so the samples scoring below were considered undetermined, and included in another plate for confirmation of the genotype. Two of us, A.D.-A. and J.O.H.-L., independently confirmed the genotypes. For *SLC16A11* s75493593 marker, we re-genotyped 12% of the samples and independently confirmed the genotypes. In a few cases, samples kept their status of undetermined, no genotype was assigned, and the samples were excluded. Markers *FTO* rs9939609, assay C_30090620_10, [Vic/Fam] [A/T], *TCF7L2* rs7895307, assay C_31976175_10 [Vic/Fam] [A/G], and *SLC16A11* rs75493593, assay C_102213324_10 [Vic] [Fam] [G/T], were analyzed with 40 cycles of 15 s at 95 °C and 90 s at 60 °C, with a single previous denaturation of 10 min at 95 °C. For *FTO* marker, genotypes were coded as AA, AT, or AT, and for a dominant model as carriers of A (AA and AT) or non-carriers (TT). For *TCF7L2* marker, AA, AG, and GG genotypes, and for the dominant model GG vs. AG/AA were considered. For *SLC16A11* marker, we considered GG, GT, and TT genotypes and the dominant model of carriers of G (GG and GT) versus non-carriers (TT).

SPSS program V.21 was used for statistical analyses. Given that we evaluated three markers, significance level was adjusted to α = 0.016. The sample size was calculated with G*Power V.3.1.9.6 for linear multiple regression, considering two tails, an effect size of 0.15, α error probability of 0.016, power of 0.8 and three predictors, in two groups and three different genetic markers. Considering the multifactorial nature of the studied phenotypes, we were expecting weak to moderate associations, so no further correction was applied to avoid missing possibly true but subtle effects. For continuous variables, descriptive statistics are presented as means and standard deviation (or medians and interquartile ranges according to the Kolmogorov–Smirnov test). No sex comparisons were made at this stage. Categorical variables are presented as frequencies and percentages. For inferential statistics, we used the listwise deletion of cases with missing values in the variables involved in each test. These analyses included bivariate nominal analyses with Chi-squared or Fisher Exact tests, and odds ratio (OR). For continuous variables, t test or ANOVA or for non-normally distributed variables, Mann–Whitney or Kruskal–Wallis tests were used. Multivariate linear regression models were used to evaluate the association between the genetic variants and metabolic parameters, including age and sex. Inspection of residual plots was carried out to define if there was no substantial departure from normality. Models were chosen according to previous reports regarding associations with each genetic marker. We also tested a previously proposed hypothesis of an association between rs7895307 and depression using logistic regression and OR.

## 5. Conclusions

This study provides evidence on the association of A allele in *FTO* gene with severe obesity according to BMI Z score, waist circumference, and muscle percentage in Mexican children with adiposity. If these results are confirmed in large samples, and accompanied by more comprehensive genetic analysis, they may contribute to highlight the importance of considering predisposing genetic factors in predicting the risk of obesity and other metabolic conditions, extending the knowledge about the genetic profile of children of understudied populations. Our results also propose the hypothesis of a higher risk for depression in adolescents who are women or who are carriers of AA genotype in *TCF7L2* gene marker.

Due to the characteristics and exploratory design of our study, research should continue to investigate these genetic correlates in large and well-characterized samples of diverse populations, including Latin American groups, which could be crucial to finally develop more effective strategies in the study of metabolic diseases.

## Figures and Tables

**Table 1:** Characteristics of the sample in Group 1.

Variables	Males (n = 83)				Females (n = 92)			
**Non-normally distributed**	**n**	**Median**	**Q1**	**Q3**	**n**	**Median**	**Q1**	**Q3**
Percentile BMI WHO	74	99.07	96.13	99.8	78	98.9	94.18	99.78
Triglycerides (mg/dL)	82	123.13	75	181.5	90	116.5	84.5	148.25
Uric Acid (mg/dL)	76	6.34	5.18	7.43	86	5.37	4.59	6.08
ALT (mg/dL)	75	22.4	12	43.48	86	17.15	12.13	28.48
AST (mg/dL)	75	22.4	17.7	30.18	86	20.5	17.1	27.1
GGT (mg/dL)	45	17.6	14	31.25	43	15.7	12.85	20.35
C-Peptide (ng/mL)	65	3	1.97	4.12	67	3.28	2.22	4.5
**Normally distributed**	**n**	**Mean**	**SD**		**n**	**Mean**	**SD**	
Age	83	14.2	3.99		91	13.61	3.69	
BMI (kg/m^2^)	83	27.79	5.96		92	27.42	5.47	
BMI Z score	73	2.29	1.07		78	2.21	0.94	
Fat (%)	82	35.57	8.64		90	39.64	7.05	
Fat (kg)	82	26.93	13.33		90	26.41	10.86	
Muscle (kg)	82	25.62	8.11		90	20.82	5.53	
Waist circumference (cm)	75	91.5	15.88		83	83.89	13.63	
Waist-to-height ratio	75	0.57	0.07		83	0.55	0.09	
HbA1c (%)	81	4.86	0.56		90	4.78	0.53	
Fasting glucose (mg/dL)	82	88.75	11.02		89	84.98	11.11	
Total cholesterol(mg/dL)	82	170.54	37.4		90	165.7	35.38	
HDL-C (mg/dL)	81	43.68	9.47		90	42.77	9.69	
LDL-C (mg/dL)	80	97.05	24.2		90	95.63	25.28	
Insulin (µU/mL)	36	21.04	14.39		48	18.38	12.8	

Q1: 25th percentile, Q3: 75th percentile; BMI: body mass index; WHO: World Health Organization; ALT: alanine aminotransferase; AST: aspartate aminotransferase; GGT: gamma-glutamyl transferase; HbA1c: hemoglobin A1c; HDL-C: High-density lipoprotein cholesterol; LDL-C: Low-density lipoprotein cholesterol.

**Table 2:** Characteristics of the sample in Group 2.

Variables	Males (n = 151)				Females (n = 145)			
**Non-normally distributed**	**n**	**Median**	**Q1**	**Q3**	**n**	**Median**	**Q1**	**Q3**
Age (years)	151	14	13	16	145	15	14	16
SBP (mmHg)	132	100	100	110	130	100	90	110
DSP (mmHg)	131	70	60	70	130	61.5	60	70
SBP Percentile	131	24	6.5	55.5	130	21	6.75	47.75
DBP Percentile	131	61.5	37	73	130	45	33.25	69
BMI Z score	148	0.48	−0.31	1.33	143	0.73	0.17	1.32
BMI percentile	148	67.95	41.2	91.3	143	77.5	56.6	91.25
Triglycerides (mg/dL)	138	91.5	59.5	125.25	131	94	69	130
**Normally distributed**	**n**	**Mean**	**SD**		**n**	**Mean**	**SD**	
Waist circumference (cm)	65	78.77	10.99		65	77.11	12.81	
Fat (%)	82	19.98	8.04		81	29.13	7.2	
Fasting glucose (mg/dL)	146	91.62	6.39		140	88.47	7.07	
Uric Acid (mg/dL)	146	5.87	1.16		140	4.56	1.23	
Total cholesterol (mg/dL)	142	147.99	27.45		131	154.91	30.22	
HDL-C (mg/dL)	49	44.94	11.04		49	44.98	11.28	
LDL-C (mg/dL)	49	87.01	27.55		49	85.92	23.38	

SBP = systolic blood pressure; DBP = diastolic blood pressure.

**Table 3:** Genotype and allele frequencies in the sample.

Marker	Genotype 1 n (%)	Genotype 2 n (%)	Genotype 3 n (%)	Allele 1 n (%)	Allele 2 n (%)	H–W X^2^ (*p* Value)
FTO rs9939609	AA	AT	TT	A	T	
	6 (3.43)	58 (33.14)	111 (63.43)	70 (20)	280 (80)	0.22 (0.64)
	14 (4.73)	97 (32.77)	185(62.50)	125 (21.11)	467 (78.89)	0.08 (0.78)
TCF7L2 rs7895307	AA	AG	GG	A	G	
	71 (40.57)	74 (42.29)	30 (17.14)	216 (61.71)	134 (38.29)	1.94 (0.16)
	113(38.7)	139 (47.6)	40 (13.7)	365 (63.48)	219 (36.52)	0.07 (0.79)
SLC16A11 rs75493593	GG	GT	TT	G	T	
	81 (46.55)	64 (36.78)	29 (16.67)	226 (64.94)	122 (35.06)	6.43 (0.011)
	112 (38.22)	122 (41.64)	59 (20.14)	346 (59.04)	240 (40.96)	5.67 (0.017)

H–W: Hardy–Weinberg equilibrium test. Group 1: first row of each marker; Group 2: second row of each marker.

**Table 4:** Predictors of metabolic parameters including rs9939609 *FTO* marker.

Model	Unstandardized Coef. Beta	Standardized Coef. Beta	Std. Error	t	*p* Value	95% CI for B Lower Bound	95% CI for B Upper Bound
**BMI Z Score in Group 1**							
**Constant**	2.56		0.38	6.81	<0.001	1.82	3.31
**Male sex**	0.12	0.06	0.16	0.78	0.438	−0.19	0.44
**Age**	−0.04	−0.12	0.03	−1.53	0.129	−0.10	0.01
***FTO* AA/AT**	0.45	0.22	0.16	2.76	0.007	0.13	0.78
**Waist Circumference in Group 1**							
**Constant**	60.19		4.37	13.77	<0.001	51.56	68.83
**Male sex**	6.63	0.22	2.15	3.08	0.002	2.38	10.89
**Age**	1.59	0.38	0.30	5.38	<0.001	1.01	2.18
***FTO* AA/AT**	6.16	0.20	2.22	2.78	0.006	1.78	10.55
**Fat percentage in Group 1**							
**Constant**	32.84		3.17	10.35	<0.001	26.57	39.11
**Female sex**	4.04	0.25	1.29	3.13	0.002	1.49	6.59
**Age**	0.14	0.05	0.22	0.65	0.52	−0.30	0.59
***FTO* AA/AT**	3.14	0.19	1.32	2.37	0.019	0.52	5.76
**Fat percentage in Group 2**							
**Constant**	31.78		6.20	5.12	<0.001	19.53	44.02
**Female sex**	9.51	0.54	1.16	8.17	<0.001	7.21	11.81
**Age**	−0.89	−0.14	0.43	−2.08	0.039	−1.74	−0.04
***FTO* AA/AT**	2.67	0.15	1.21	2.21	0.029	0.28	5.06
**Muscle percentage in Group 1**							
**Constant**	−21.36		3.64	−5.88	<0.001	−28.61	−14.12
**Male Sex**	6.00	0.28	1.61	3.73	<0.001	2.79	9.21
**Age**	2.67	0.71	0.28	9.68	<0.001	2.12	3.22
***FTO* AA/AT**	4.21	0.19	1.66	2.54	0.013	0.91	7.51

Coef: coefficient; Std: standard.

**Table 5:** Genotype and allele frequencies according to HOMA-IR index in Group 1.

Genotype	Insulin Sensitivity (≤2.8) n (%)	Insulin Resistance (≥2.9) n (%)	Allele	Insulin Sensitivity (≤2.8) n (%)	Insulin Resistance (≥2.9) n (%)
*FTO* rs9939609					
AA	4 (9.52)	0 (0) ^a^	A	20 (23.81)	15 (15.63) ^b^
AT	12 (28.57)	15 (31.25)	T	64 (76.19)	81 (84.37)
TT	26 (61.91)	33 (68.75)			
*TCF7L2* rs7895307					
AA	19 (45.24%)	15 (31.25) ^c^	A	58 (69.05%)	50 (52.08) ^d^
AG	20 (47.62%)	20 (41.67)	G	26 (30.95%)	46 (47.92)
GG	3 (7.14%)	13 (27.08)			
*SLC16A11* rs75493593					
GG	15 (35.72)	25 (53.19) ^e^	G	44 (52.38)	68 (72.34) ^f^
GT	14 (33.33)	18 (38.30)	T	40 (47.62)	26 (27.66)
TT	13 (30.95)	4 (8.51)			

HOMA-IR: Homeostatic Model Assessment for Insulin Resistance. ^a^*FTO* AA/AT vs. TT Pearson χ^2^ = 0.46, OR = 0.74, 95% CI [0.31–1.77], *p* = 0.50; ^b^ *FTO* A vs. T allele Pearson χ^2^ = 1.92, OR = 0.59, 95% CI [0.28–1.25], *p* = 0.17; ^c^ *TCF7L2* AA/AG vs. GG OR = 0.21, 95% CI [0.05–0.79], Fisher *p* = 0.03; ^d^ *TCF7L2* A vs. G allele Pearson χ^2^ = 5.37, OR = 0.49, 95% CI [0.26–0.90], *p* = 0.014; ^e^ *SLC16A11* GG/GT vs. TT Fisher *p* = 0.013, OR = 4.82, 95% CI [1.43–16.25]; ^f^ *SLC16A11* G vs. T allele Pearson χ^2^ = 7.57, OR = 2.37, 95% CI [1.28–4.43], *p* = 0.005.

**Table 6:** Predictors of metabolic parameters including rs7895307 *TCF7L2* marker.

Model	Unstandardized Coef. Beta	Standardized Coef. Beta	Std. Error	t	*p* Value	95% CI for B Lower Bound	95% CI for B Upper Bound
**Glucose in Group 1**							
**Constant**	77.27		4.49	17.20	<0.001	68.39	86.15
**Female sex**	−3.28	−0.15	1.76	1.87	0.064	−6.75	0.19
**Age**	0.72	0.19	0.31	2.35	0.02	0.11	1.33
**TCF7L2 GA**	2.98	0.13	1.93	1.54	0.126	−0.84	6.81
**TCF7L2 GG**	6.05	0.21	2.50	2.42	0.017	1.11	10.99
**Glucose in Group 2**							
**Constant**	92.26		4.06	22.74	<0.001	84.27	100.25
**Male sex**	3.13	0.23	0.79	3.95	<0.001	1.57	4.70
**Age**	−0.25	−0.05	0.27	−0.92	0.359	−0.79	0.29
**TCF7L2 GA**	0.37	0.03	0.85	0.44	0.659	−1.29	2.04
**TCF7L2 GG**	−1.90	−0.09	1.28	−1.49	0.137	−4.41	0.61

**Table 7:** Predictors of metabolic parameters including rs75493593 *SLC16A11* marker.

Fasting Glucose in Group 2							
Model	Unstandardized Coef. Beta	Standardized Coef. Beta	Std. Error	t	*p*	95% CI for B Lower Bound	95% CI for B Upper Bound
**Constant**	89.48		4.40	20.36	<0.001	80.83	98.14
**Male sex**	3.55	0.25	0.76	4.70	<0.001	2.06	5.03
**Age**	−0.35	−0.07	0.26	−1.35	0.179	−0.85	0.16
**BMI percentile**	0.03	0.12	0.01	2.26	0.024	0.00	0.06
**SLC16A11 GG/GT**	1.97	−0.13	0.80	2.47	0.014	0.40	3.54

## Data Availability

The original contributions presented in this study are included in the article. Further inquiries can be directed to the corresponding authors. Data will be available to interested researchers upon request.
